# Fearful contextual expression impairs the encoding and recognition of target faces: an ERP study

**DOI:** 10.3389/fnbeh.2015.00237

**Published:** 2015-09-02

**Authors:** Huiyan Lin, Claudia Schulz, Thomas Straube

**Affiliations:** Institute of Medical Psychology and Systems Neuroscience, University of MuensterMuenster, Germany

**Keywords:** facial expression, ERP, emotional context, encoding, recognition, N170

## Abstract

Previous event-related potential (ERP) studies have shown that the N170 to faces is modulated by the emotion of the face and its context. However, it is unclear how the encoding of emotional target faces as reflected in the N170 is modulated by the preceding contextual facial expression when temporal onset and identity of target faces are unpredictable. In addition, no study as yet has investigated whether contextual facial expression modulates later recognition of target faces. To address these issues, participants in the present study were asked to identify target faces (fearful or neutral) that were presented after a sequence of fearful or neutral contextual faces. The number of sequential contextual faces was random and contextual and target faces were of different identities so that temporal onset and identity of target faces were unpredictable. Electroencephalography (EEG) data was recorded during the encoding phase. Subsequently, participants had to perform an unexpected old/new recognition task in which target face identities were presented in either the encoded or the non-encoded expression. ERP data showed a reduced N170 to target faces in fearful as compared to neutral context regardless of target facial expression. In the later recognition phase, recognition rates were reduced for target faces in the encoded expression when they had been encountered in fearful as compared to neutral context. The present findings suggest that fearful compared to neutral contextual faces reduce the allocation of attentional resources towards target faces, which results in limited encoding and recognition of target faces.

## Introduction

Several studies have investigated whether the expression of a face influences its encoding (e.g., Batty and Taylor, 2003; Blau et al., 2007; Frühholz et al., 2011) and recognition (e.g., Johansson et al., 2004; Wagner et al., 2007; Satterthwaite et al., 2009; Sessa et al., 2011; Righi et al., 2012; Pinabiaux et al., 2013), at least when faces are presented in isolation. Face encoding is thought to be reflected by the N170, a face-sensitive Event-Related Potential (ERP) component which peaks around 170 ms after stimulus onset and is maximal at occipito-temporal scalp sites (e.g., Bruce and Young, 1986; Eimer, 2000; Bentin et al., 2007). During the encoding of emotional faces, Batty and Taylor (2003) reported larger N170 amplitudes to fearful as compared to neutral, happy, disgusted, surprised, sad and angry faces. The increased N170 to fearful faces was suggested to be the result of mobilization of attention, which might enhance face encoding. For face recognition, expression effects differ according to whether participants are explicitly asked to memorize the facial identities during the encoding phase (explicit recognition) or not (implicit recognition), and to whether faces are presented in the encoded expressions or a neutral expression during the recognition phase. In explicit recognition, fearful as compared to neutral facial identities were better recognized when these identities showed the encoded expression during the recognition phase (Sessa et al., 2011; Pinabiaux et al., 2013). When faces that had been encoded in a different expression were presented with a neutral expression during the recognition phase, Righi et al. (2012) found enhanced recognition performance for fearful-as compared to happy-encoded faces. In implicit recognition, it is still unclear whether facial expressions modulate recognition performance of facial identities when the identities showed the encoded expression during the recognition phase. Specifically, Johansson et al. (2004) did not find an effect of facial expression on recognition performance. However, there was a trend for the effect of facial expression in Wagner et al. (2007) study. When emotionally encoded faces are presented with a neutral expression during the recognition phase, Satterthwaite et al. (2009) did not find an effect of the encoded expression (threat vs. non-threat) on recognition performance.

However, in many circumstances, relevant faces do not appear in isolation but in a context of other emotional stimuli, such as emotional pictures and other emotional faces. In this field of research, several previous studies have investigated the emotional effect of concomitant contextual pictures on face encoding and recognition (e.g., Galli et al., 2006; Righart and de Gelder, 2006, 2008a,b; Van den Stock and de Gelder, 2012). For instance, during the encoding phase, emotional (fearful and happy) faces presented in fearful scenes as compared to these faces presented in happy or neutral scenes were found to evoke larger N170 amplitudes (Righart and de Gelder, 2008a). In addition, neutral faces that had been encoded together with emotional pictures (e.g., scenes and bodies) were found to be recognized to a lesser degree than these neutral faces when they had been encoded together with neutral pictures (Van den Stock and de Gelder, 2012).

Meanwhile, two electroencephalography (EEG) studies investigated the effect of preceding contextual facial expression on facial encoding (Furl et al., 2007; Richards et al., 2013). In a Magnetoencephalography (MEG) study, Furl et al. (2007) found that a sequence of fearful contextual faces as compared to a sequence of neutral contextual faces reduced the M170 (which is thought to be the MEG counterpart of the N170; Deffke et al., 2007) to an upcoming fearful or neutral face when only one target face followed the contextual faces. However, an ERP study by Richards et al. (2013) did not report effects of preceding contextual facial expression on the N170 to target faces when several target faces were presented after the contextual faces. The discrepancy might be due to the number ratio between contextual and target faces. The ratio was large in Furl et al. (2007) study as the number was much larger for contextual compared to target faces (20:1), but small in Richards et al. (2013) as the number was similar between contextual and target faces (35:33). The reduced ratio between contextual and target faces may eliminate the effect of contextual facial expression on the N170 to target faces.

In the above-mentioned studies, the sequence of contextual and target faces is fixed (i.e., one or a fixed number of target face(s) always followed a fixed number of contextual faces), and the temporal onset of target faces was perfectly predictable. However, it is still unclear what the effect of contextual facial expression on target faces would be when the temporal onset of target faces is unpredictable. Furthermore, these two studies basically investigated effects of adaptation. Adaptation in these cases refers to repeating one identity with one expression to modulate the perception of this identity showing the same or another expression. In this case, the contextual and the target face shared the same identity and participants could predict the identity of target faces. However, if participants cannot predict the identity of target faces, how does contextual facial expression then influence the encoding of the target faces? How do ERP effects look like if identity *and* expression vary?

Moreover, while previous studies have already investigated the effect of preceding contextual facial expression on the encoding of target faces, no study as yet has investigated whether contextual facial expression modulates later recognition of target faces. More importantly, facial expressions are always changing; we seldom see a person showing the same expression when we meet him/her for the second time. Therefore, it is also interesting to determine whether contextual facial expression modulates recognition of target faces when target faces show non-encoded expressions.

In the present study, we aimed to investigate whether the encoding and recognition of emotional target faces depend on preceding contextual facial expressions. To this end, participants had to indicate the presence of target faces (fearful or neutral) that were shown after a sequence of fearful or neutral contextual faces. The number of contextual faces in a sequence was random so that the temporal onset of target faces was unpredictable. Contextual and target faces were of different identities so that participants could not predict the identities of the target faces. In addition, EEG was recorded during the encoding phase. Subsequently, participants had to perform an old/new recognition task in which target faces were presented in either the encoded or a non-encoded expression. The recognition task was unexpected and participants did not know about the task until the end of the encoding phase in order not to influence the initial encoding of target faces. In line with previous studies, we expected that if contextual facial expression modulated the encoding and recognition of target faces, fearful as compared to neutral contextual expression would reduce N170 amplitudes for target faces during the encoding phase and subsequently, recognition performance (e.g., accuracy, ACC) during the recognition phase. In addition, considering the emotional effect of target faces, we also expected that fearful as compared to neutral target faces would elicit larger N170 amplitudes during the encoding phase and would be better recognized during the recognition phase, at least when target faces showed the encoded expression.

## Materials and Methods

### Participants

Twenty-four participants were recruited in Muenster via advertisement, and were paid 10 Euros for participation. Two participant were excluded from the statistical analysis because of excessive artifacts in the EEG signal, resulting in a total of 22 participants (19–35 years, *M* = 25.57, *SD* = 4.91; 15 females). All participants were right-handed as determined by the Edinburgh Handedness Inventory (Oldfield, 1971). Participants had normal and corrected-to-normal vision and no participants reported a history of neurological illness. All participants gave written informed consent in accordance with standard ethical guidelines as defined in the Declaration of Helsinki. The study was approved by the ethics committee of University of Muenster.

### Stimuli

We selected 488 digitized color pictures from FACES (Ebner et al., 2010), Karolinska Directed Emotional Faces (KDEF; Lundqvist et al., 1998), NimStim (Tottenham et al., 2009) and Radboud Faces Database (RaFD; Langner et al., 2010). Facial pictures portrayed 244 identities (122 males and 122 females) with a fearful and a neutral expression each. As facial pictures were derived from different databases, they were cropped similarly around the face outline and centered so that eyes, nose and mouth were at similar positions for all faces. Non-facial parts (e.g., neck, shoulders and distant hair) were removed. Facial pictures were adjusted to a size of 7.49 cm (horizontal) × 9.99 cm (vertical), and aligned in luminance, contrast, hue and color using Adobe Photoshop CS6. Finally, the background color was set to black.

Of these facial pictures, four pictures of two identities (1 male and 1 female; 2 fearful and 2 neutral) served as contextual pictures in the encoding phase. 244 facial pictures (including 4 practice items) of 122 identities (61 males and 61 females; 122 neutral, 122 fearful) were used as target facial pictures. The remaining 240 pictures of 120 identities (60 males and 60 females; 120 fearful and 120 neutral) were novel facial pictures during the recognition phase.

For target faces used in the actual experiment, stimuli were separated into 4 sets pseudo-randomly with 30 identities (15 females and 15 males; 30 fearful and 30 neutral) each. Each set was separated into two sub-sets (30 identities each) according to the expressions. For the encoding phase, half of the sub-sets with different identities were used to create four experimental conditions for the encoding phase: fearful targets among fearful context faces, fearful targets among neutral context faces, neutral targets among fearful context faces and neutral targets among neutral context faces. Assignment of sub-sets was counterbalanced across participants. For the recognition phase, both sub-sets were presented to have two conditions for learned faces: changed and unchanged (with respect to expression). Novel identities in the recognition phase were also presented with the two expressions.

### Procedure

After giving written informed consent, participants were seated at a viewing distance of approximately 80 cm from a 15-inch CRT computer screen. Stimulus presentation was controlled with E-Prime 2.0 software (Psychology Software Tools, Inc., Pittsburgh, PA, USA), and the screen resolution was 1280 by 1024 pixel, thus resulting in face stimulus presentation of 5.37° × 7.15° of visual angle.

Prior to the encoding phase, participants had to familiarize themselves with the four contextual faces. Participants were then told that they would be presented with these four faces (contextual faces) and some other ones (target faces) and that they had to indicate whether the prompted face was a contextual or a target one by pressing the “F” or the “J” key with their left and right index finger, respectively. The instructions emphasized speed as well as ACC. Response assignments were counterbalanced across participants. Practice trials in which feedback on ACC and response times (RT) was provided were performed prior to the actual experiment in order to ensure that participant memorized the contextual faces and were familiarized with the experimental procedure. Each trial started with a white fixation cross which was presented for 200 ms, followed by a blank screen for 600–1000 ms (*M* = 800 ms). Subsequently, either a contextual or a target face was presented for 800 ms. The presentations of contextual and target faces were completely randomized. Another blank screen was presented for 1000 ms before the next trial started. Button presses were allowed during the presentation of the face or the following blank. Facial pictures were presented separately in four different blocks according to the expression of contextual and target faces (fearful target among fearful context, fearful target among neutral context, neutral target among neutral context, and neutral target among fearful context). Block sequence was randomized across participants. For a block, each target facial picture was presented twice, and each contextual facial picture was presented 90 times. Therefore, the encoding phase of the experiment consisted of a total of 960 (30 × 2 × 4 + 2 × 90 × 4) trials.

The recognition phase followed the encoding phase after approximately 5 min. Participants were just informed about the recognition task at this point in the experiment. Participants were asked to indicate whether a prompted facial identity had been presented in the preceding encoding phase (target/old identity) or not (novel identity) by pressing “F” or “J” key. It was particularly emphasized that the old/new judgment was based on facial identity, regardless of facial expression (e.g., “Identity A previously showed a fearful expression. Now, A will either show a fearful or a neutral expression. Both fearful and neutral A should be judged as ‘old’ ”). The instructions emphasized speed as well as ACC. Response assignments were counterbalanced across participants. Each trial started with a white fixation cross presented for 200 ms, followed by a blank screen for 600–1000 ms (*M* = 800 ms). Subsequently, either a target face or a novel face was presented for 500 ms. The next trial started after the presentation of another blank screen for 1000 ms. Button presses were allowed during the presentation of the face or the following blank. Each facial picture was presented once, resulting in 480 (240 + 240) trials in total. The complete experiment including the encoding and the recognition phase lasted about 1.5 h.

### Behavior Recording

For the encoding and the recognition phase, ACC and RT of button presses in the time range from the onset of the target face to the offset of the following blank were recorded. For the analysis of RT, trials only with correct responses were included.

### EEG Recording

Continuous EEG was recorded using a 32-channel BioSemi ActiveTwo system (BioSemi, Amsterdam, Netherlands) from 32 Ag/AgCl active electrodes. These electrodes were attached to an elastic cap (BioSemi, Amsterdam) and arranged according to the international extended 10–20 system (FP1, FP2, F7, F3, Fz, F4, F8, FC5, FC1, FC2, FC6, C3, Cz, C4, T7, T8, TP9, TP10, CP1, CP2, P7, P9, P3, Pz, P4, P8, P10, PO9, PO10, O1, Oz, O2). The BioSemi system uses a combined ground and reference circuit with an active electrode (common mode sense, CMS) and a passive electrode (driven right leg, DRL) (please refer to http://www.biosemi.com/faq/cms&drl.htm for more details). To monitor eye blinks and movements, horizontal electrooculogram (EOG) was recorded from two electrodes at the outer canthi of both eyes and vertical EOG was recorded bipolarly from two electrodes above and below the right eye. All signals were recorded in DC mode and digitized with a sampling rate of 2048 Hz.

Offline, ocular artifacts were automatically inspected and corrected by using BESA 6.0 software.^1^ EEG data were segmented into 1000 ms epochs from −200 to 800 ms relative to the onset of the target faces (regardless of response ACC), with the first 200 ms epochs for baseline correction. Thresholds for artifact rejection were and amplitude threshold of 120 μV, a gradient criterion of 75 μV and low signal criterion of 0.01 μV (default parameters of the BESA 6.0 artifact rejection tool). Artifact-free trials were then averaged separately for each electrode and each experimental condition. Averaged ERPs were recalculated to average reference, excluding vertical and horizontal EOG channels. ERPs were low pass filtered (40 Hz, Butterworth, zero phase shift).

ERPs were quantified by mean amplitudes for N170 (135–175 ms) relative to −200 ms baseline. The N170 was measured at electrodes PO9 and PO10. The time window for the N170 was chosen according to the peak latency identified in the grand waveforms across all conditions (155 ms). Electrodes of interest were based on visual inspection of the grand waveforms and previous studies (Latinus and Taylor, 2006; Righart and de Gelder, 2006, 2008a; Herbert et al., 2013).

It should be noted that EEG was also recorded during the recognition phase. However, we could not further analyze the ERPs for these faces due to insufficient numbers of correct response trials (on average about 10 per experimental condition).

### Data Analysis

#### Encoding Phase

For statistical analyses of behavioral data, RT and ACC for target faces during the encoding phase were entered into 2 × 2 repeated measures analyses of variance (ANOVAs) with “contextual expression” (fearful vs. neutral) and “target expression” (fearful vs. neutral) as within-subject factors. For statistical analysis of ERP data, the additional within-subject factor hemisphere (left vs. right) was included. Degrees of freedom and *p* values of ANOVAs were corrected using Greenhouse-Geisser correction where necessary.

#### Recognition Phase

RT and ACC for the learned target faces during the recognition phase were entered into 2 × 2 × 2 repeated measures ANOVAs with “consistency” (unchanged vs. changed), “contextual expression” (fearful vs. neutral) and “encoded target expression” (fearful vs. neutral) as within-subject factors. Degrees of freedom and *p* values of ANOVAs were corrected using Greenhouse-Geisser correction where necessary.

## Results

### Encoding Phase

#### Behavioral Data

**Figure 1:**
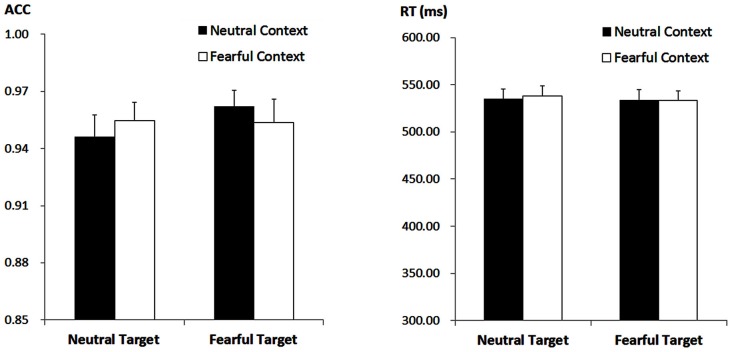
**Accuracy (ACC; Left panel) and response times (RT, Right panel) for all the experimental conditions during the encoding phase**. Vertical lines indicate the standard error of the mean.

The ANOVA on ACC only revealed a significant main effect of “target expression” (*F*_(1,21)_ = 5.47, *p* = 0.029, ${\text{η}}_{\text{p}}^{2}$ = 0.21), with higher ACC for fearful (*M ± SE* = 95.80 ± 0.93%) as compared to neutral target faces (95.04 ± 0.99%). Other effects failed to reach statistical significance (*ps* > 0.05). For the RT, the analysis failed to reveal any significant main effects or interactions (*ps* > 0.10) Figure 1.

#### ERP Data—N170

**Figure 2:**
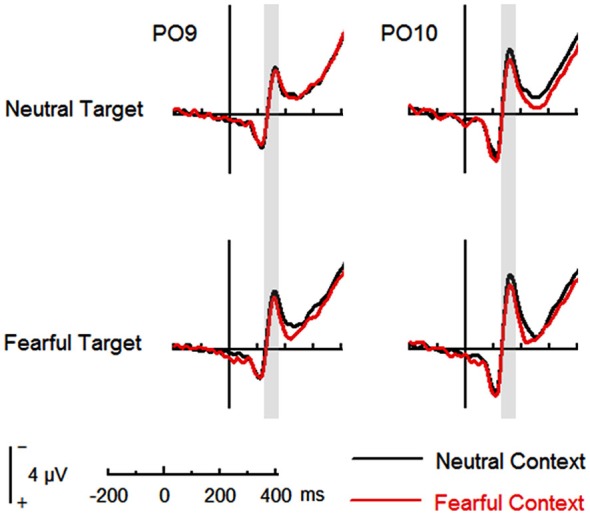
**Event-related potentials (ERPs) at parieto-occipital electrodes (PO9 and PO10) for all the experimental conditions during the encoding phase**. Shaded areas correspond to the time window for the N170 analysis.

**Figure 3:**
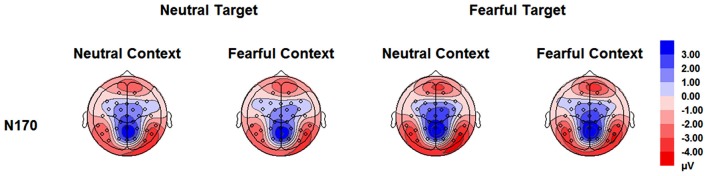
**Topographical maps based on N170 mean amplitudes for all the experimental conditions during the encoding phase**.

The analysis on N170 showed significant main effects of “contextual expression” (*F*_(1,21)_ = 4.53, *p* = 0.045, ${\text{η}}_{\text{p}}^{2}$ = 0.18), “target expression” (*F*_(1,21)_ = 15.40, *p* = 0.001, ${\text{η}}_{\text{p}}^{2}$ = 0.42) and hemisphere (*F*_(1,21)_ = 4.64, *p* = 0.043, ${\text{η}}_{\text{p}}^{2}$ = 0.18). Overall, the N170 amplitudes were significantly larger for target faces after a sequence of neutral contextual faces (−3.30 ± 0.54 μV) as compared to target faces after a sequence of fearful contextual faces (−2.93 ± 0.54 μV), for fearful (−3.46 ± 0.53 μV) as compared to neutral target faces (−2.77 ± 0.55 μV) and over the right (−3.62 ± 0.68 μV) as compared to the left hemisphere (−2.61 ± 0.46 μV). However, the analysis did not show any interactions of these factors (*ps* > 0.10) Figures 2, 3 and Table 1.

**Table 1:** **Mean amplitudes (μV) of N170 and its standard error for all the experimental conditions**.

	Context			
	Neutral		Fearful	
Target	PO9	PO10	PO9	PO10
Neutral	−2.27 (0.48)	−3.58 (0.70)	−2.24 (0.53)	−2.97 (0.70)
Fearful	−3.14 (0.53)	−4.21 (0.71)	−2.79 (0.44)	−3.71 (0.72)

### Recognition Phase

#### Accuracy

The analysis showed a main effect of “consistency” (*F*_(1,21)_ = 5.29, *p* = 0.032, ${\text{η}}_{\text{p}}^{2}$ = 0.20). Response ACC was higher for target faces showing the encoded expression (40.64 ± 3.42%) as compared to target faces showing the non-encoded expression (37.05 ± 3.18%). However, this effect was modulated by interactions with “contextual expression” (*F*_(1,21)_ = 5.55, *p* = 0.028, ${\text{η}}_{\text{p}}^{2}$ = 0.21) as well as with “encoded target expression” (*F*_(1,21)_ = 7.89, *p* = 0.011, ${\text{η}}_{\text{p}}^{2}$ = 0.27).

When target faces were presented with the encoded expression, ACC was higher for those that had been encoded after neutral contextual faces (42.05 ± 3.45%) than after fearful contextual faces (39.24 ± 3.50%; *F*_(1,21)_ = 4.86, *p* = 0.039, ${\text{η}}_{\text{p}}^{2}$ = 0.19). However, when target faces changed the expression, there was no significant effect of the contextual expression anymore (*F*_(1,21)_ = 0.50, *p* = 0.487, ${\text{η}}_{\text{p}}^{2}$ = 0.023; 36.44 ± 3.32% vs. 37.65 ± 3.26%).

**Figure 4:**
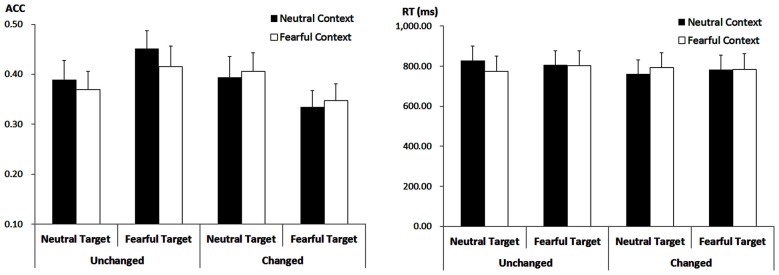
**ACC (Left panel) and RT (Right panel) for all the experimental conditions during the recognition phase**. Vertical lines indicate the standard error of the mean.

Moreover, when target faces showed the encoded expression, ACC was higher for those faces encoded and recognized as fearful (43.33 ± 3.75%) as compared to those encoded and recognized as neutral (37.95 ± 3.53%; *F*_(1,21)_ = 4.55, *p* = 0.045, ${\text{η}}_{\text{p}}^{2}$ = 0.18). When target faces showed the non-encoded expression, however, the ACC was lower for those faces encoded as fearful and now shown as neutral (34.09 ± 3.09%) as compared to those encoded as neutral but now shown with a fearful expression (40.00 ± 3.70%; *F*_(1,21)_ = 5.74, *p* = 0.026, ${\text{η}}_{\text{p}}^{2}$ = 0.22). Other main effects or interactions failed to reach statistical significance (*ps* > 0.10; Figure 4).

While the analysis showed enhanced ACC for fearful target faces during the recognition phase regardless of the encoded emotion, the analysis of ACC values cannot rule out yet whether this facial expression effect was due to a negativity bias in which negative as compared to neutral stimuli are more likely to be judged as “old” (e.g., Sharot et al., 2004). In order to investigate this issue we computed discrimination measure (Pr), as Pr provides an unbiased estimate of the ACC by taking responses to new items into account (e.g., Snodgrass and Corwin, 1988). Of note, Pr of fearful faces and that of neutral faces were used all fearful and neutral target faces during the recognition phase, respectively, regardless of the encoded expression in order to match the number between old/target and new faces. The repeated measured ANOVA with “expression during the recognition phase” (fearful vs. neutral) clearly showed that the above-described facial expression effect were driven by a negativity bias, as target faces showing fearful expression during the recognition phase and the faces showing neutral expression did not differ in Pr (*ps* > 0.10).

#### Response Times

The analysis on the target faces only showed a trend for the three-way interaction (*F*_(1,21)_ = 3.75, *p* = 0.066, ${\text{η}}_{\text{p}}^{2}$ = 0.15). For fearful-encoded faces, we did not find any main effects or interaction (*ps* > 0.10). For neutral-encoded faces, while there were no main effects of “consistency” and “contextual expression” (*ps* > 0.10), their interaction was significant (*F*_(1,21)_ = 5.01, *p* = 0.036, ${\text{η}}_{\text{p}}^{2}$ = 0.19). When these target faces showed the encoded expression, there was only a trend for the effect of “contextual expression” (*F*_(1,21)_ = 3.30, *p* = 0.084, ${\text{η}}_{\text{p}}^{2}$ = 0.14), with slightly longer RT for target faces that had been encoded after neutral contextual faces (827.12 ± 73.43 ms) than after fearful contextual faces (773.92 ± 74.92 ms). When these target faces showed the non-encoded expression, the effect of “contextual expression” was not significant (*ps* > 0.10). Other main effects or interactions failed to reach statistical significance (*ps* > 0.10; Figure 4).

## Discussion

In this study, we investigated whether fearful as compared to neutral contextual expressions modulate the encoding and recognition of emotional target faces in an incidental learning paradigm. Results showed that during the encoding phase, fearful as compared to neutral contextual expression reduced N170 amplitudes for target faces regardless of facial expression of target faces. This context effect carried on to the recognition phase, where the ACC was lower for those target faces that had been encoded after fearful as compared to neutral contextual expression, but only when target faces showed the encoded expression. This context effect was not present when target faces showed the non-encoded expression. In addition, the N170 was larger for fearful as compared to neutral target faces during the encoding phase.

### The Effect of Contextual Facial Expression on the Encoding of Target Faces

Although contextual facial expression did not affect the behavioral classification of target faces during the encoding phase, it had a clear effect on the corresponding ERP. The finding of reduced N170 to target faces by fearful contextual expression suggests that fearful as compared to neutral contextual expression reduces the encoding of target faces (e.g., Bruce and Young, 1986; Eimer, 2000; Bentin et al., 2007). This finding is in accordance with a previous MEG study (Furl et al., 2007), which showed reduced M170 to the target face following fearful contextual faces as compared to the same target face following neutral contextual faces. It has to be noted that Furl et al. (2007) study essentially investigated the effect of adaptation. Adaptation in their study referred to the repetition of one identity showing an expression to alter the perception of this identity showing the same or another expression. In this case, the contextual and the target face were of the same identity. However, our results indicate that the adaptation account may be insufficient to explain the effect of contextual facial expression on the encoding of target faces, as contextual faces and target faces did not share the same identity in the present study. It seems to be more plausible to explain this effect in the context of available attentional resources. Flaisch et al. (2008) suggested that preceding stimuli still occupy attentional resources during presentation of target stimuli; moreover, as available attentional resources for target faces are limited and emotional as compared to neutral stimuli generally occupy more attentional resources, attention is reduced for target stimuli following emotional as compared to neutral stimuli. In addition, the N170 has been found to reflect attention during face encoding, with larger amplitudes for faces that are attended as compared to faces that are unattended (e.g., Eimer, 2000; Holmes et al., 2003; Wronka and Walentowska, 2011). Taken together, preceding fearful as compared to neutral contextual faces in the present study may occupy more attentional resources during presentation of target faces, resulting in reduced attentional resources for the encoding of target faces.

While our findings are consistent with Furl et al. (2007) study, despite our divergent interpretation of the results, the present findings are inconsistent with the study of Richards et al. (2013) which did not report an effect of contextual facial expression on the N170 to target faces. Discrepancies among Furl et al. (2007), Richards et al. (2013) and our findings may be due to different number ratios between contextual and target faces. Ratio was higher in (Furl et al. (2007); contextual vs. target faces = 20:1) and our (3:1) study as compared to Richards et al. (2013) study (35:33). During the encoding of target faces, individuals may focus much more on processing target faces as compared to preceding contextual faces. The available attentional resources to preceding contextual faces are limited. The allocation of attentional resources to preceding contextual faces may be even more limited when the ratio between contextual and target faces is low, resulting in eliminating the differential effect of contextual facial expression.

### The Effect of Contextual Facial Expression on Recognition of Target Faces

A new and important finding in the present study was that fearful contextual expression reduced later recognition rates for target faces when target faces showed the encoded expression. As described above, fearful as compared to neutral contextual faces reduced the encoding of target faces indexed by N170. Encoding, which is thought to be one of the first processes in face perception (Bruce and Young, 1986), should affect later processes of face memory, such as recognition. In line with this assumption, some ERP studies on intentional face learning indicated enhanced N170 amplitudes during encoding for those (distinctive, i.e., more visually salient) faces that would be better recognized afterwards (Schulz et al., 2012; Kaufmann et al., 2013). Accordingly, in the present study, fearful as compared to neutral contextual expression might have reduced the encoding of target faces and as a consequence, impaired recognition of target faces when target faces showed the encoded expression. However, this effect did not generalize to the non-encoded expression, possibly due to the difficulty of this incidental learning task.

For recognition of target faces showing the encoded expression, our findings might be in accordance with a study in which target faces were presented with concomitant contextual pictures (Van den Stock and de Gelder, 2012). In this study, recognition rates were found to be smaller for neutral target faces presented together with emotional pictures (e.g., bodies and scenes) as compared to the same faces presented with neutral pictures. The authors suggested that the encoding of target faces is impaired by emotional pictures. Thus, similar mechanisms may underlie effects of contextual emotion on face recognition in methodologically completely different studies.

### The Effect of Target Facial Expression on the Encoding and Recognition of Target Faces

In addition, our results showed that fearful as compared to neutral target faces evoked larger N170 amplitudes during the encoding phase, which is consistent with previous studies (e.g., Batty and Taylor, 2003; Blau et al., 2007). During the recognition phase, when faces showed the encoded expression, we replicated the results of previous studies that showed better recognition for fearful as compared to neutral faces presented in the same emotion during the encoding and the recognition phase (e.g., Sessa et al., 2011). However, this effect might be also explained by a negativity bias in which negative as compared to neutral stimuli are more likely to be judged as “old”, as follow-up analysis revealed that target faces showing fearful expression during the recognition phase and the faces showing neutral expression did not differ in Pr. It should be noted that the sensitivity of Pr has been suggested to be reduced in an unexpected recognition task (Satterthwaite et al., 2009). Therefore, further studies may use intentional learning tasks to investigate this issue in more detail.

## Limitations and Future Directions

The present study only investigated the combination of two contextual facial expressions (fearful and neutral). Further studies might also investigate whether the encoding and recognition of emotional target faces are modulated by other negative or positive contextual expressions. Furthermore, the threshold ratio relationship between contextual and target faces remains unclear. Further studies should systematically vary the ratio between contextual and target faces to investigate how the ratio between contextual and target faces modulates the effect of contextual facial expression. It is also desirable to have a higher number of correctly recognized faces to analyze corresponding ERPs during the recognition phase.

## Conclusion

The present findings indicate that the encoding and recognition of emotional target faces are modulated by preceding contextual facial expression. During the encoding phase, fearful as compared to neutral contextual expression reduced N170 amplitudes for target faces regardless of target facial expression. In the later recognition phase, while contextual facial expression did not alter recognition rates for target faces when target faces showed the non-encoded expression, fearful as compared to neutral contextual expression impaired recognition of target faces when target faces showed the encoded expression. Taken together, our findings indicate that fearful as compared to neutral contextual expression reduces available attentional resources for target faces, resulting in limited encoding and recognition of target faces.

## Author Contributions

TS and CS were involved in study design and manuscript revises. HL was involved in study design, execution, data analysis and manuscript drafting and revises. We have read and approved the final version of the manuscript together and agree to be accountable for all aspects of the work in ensuring that questions related to the accuracy or integrity of any part of the work are appropriately investigated and resolved.

## Conflict of Interest Statement

The authors declare that the research was conducted in the absence of any commercial or financial relationships that could be construed as a potential conflict of interest.

## Footnotes

^1^www.BESA.de
